# Synthesis and Structure of Methylsulfanyl Derivatives of Nickel Bis(Dicarbollide) †

**DOI:** 10.3390/molecules24244449

**Published:** 2019-12-04

**Authors:** Sergey A. Anufriev, Kyrill Yu. Suponitsky, Oleg A. Filippov, Igor B. Sivaev

**Affiliations:** 1 A.N. Nesmeyanov Institute of Organoelement Compounds, Russian Academy of Sciences, 28 Vavilov Str., Moscow 119991, Russia; trueman476@mail.ru (S.A.A.); kirshik@yahoo.com (K.Y.S.); h-bond@ineos.ac.ru (O.A.F.); 2 Shemyakin-Ovchinnikov Institute of Bioorganic Chemistry, Russian Academy of Sciences, 16/10 Miklukho-Maklay Str., Moscow 117997, Russia; 3 Basic Department of Chemistry of Innovative Materials and Technologies, G.V. Plekhanov Russian University of Economics, 36 Stremyannyi Line, Moscow 117997, Russia

**Keywords:** metallacarboranes, nickel bis(dicarbollide), SMe derivatives, synthesis, structure, hydrogen bonds, chalcogen bonds

## Abstract

Symmetrically and unsymmetrically substituted methylsulfanyl derivatives of nickel(III) bis(dicarbollide) (Bu_4_N)[8,8′-(MeS)_2_-3,3′-Ni(1,2-C_2_B_9_H_10_)_2_], (Bu_4_N)[4,4′-(MeS)_2_-3,3′-Ni(1,2-C_2_B_9_H_10_)_2_], and (Bu_4_N)[4,7′-(MeS)_2_-3,3′-Ni(1,2-C_2_B_9_H_10_)_2_] were synthesized, starting from [Ni(acac)_2_]_3_ and the corresponding methylsulfanyl derivatives of *nido*-carborane (Bu_4_N)[10-MeS-7,8-C_2_B_9_H_11_] and (Bu_4_N)[10-MeS-7,8-C_2_B_9_H_11_]. Structures of the synthesized metallacarboranes were studied by single-crystal X-ray diffraction and quantum chemical calculations. The symmetrically substituted 8,8′-isomer adopts *transoid* conformation stabilized by two pairs of intramolecular C–H···S hydrogen bonds between the dicarbollide ligands. The unsymmetrically substituted 4,7′-isomer adopts *gauche* conformation, which is stabilized by two nonequivalent C–H···S hydrogen bonds and one short chalcogen B–H···S bond (2.53 Å, −1.4 kcal/mol). The *gauche* conformation was found to be also preferred for the 4,7′-isomer.

## 1. Introduction

Molecular switches are molecules or supramolecular assemblies that can exist in two or more stable states that differ in the mutual orientation of the components and which can be transformed from one state to another by means of various external stimuli via rotation of these components relative to each other [1]. Molecular switches are the basic structural elements of any molecular electronic devices, in particular, molecular logic gates, which are capable of performing all the simplest logical and arithmetic operations, such as s summation and subtraction, encoding and decoding functions, and so on [2,3,4,5,6].

Fifteen years ago, nickel bis(dicarbollides) [3,3′-Ni(1,2-C_2_B_9_H_11_)_2_]^−1/0^ were proposed as the main construction element of molecular switches in which switching between two stable forms is achieved by reversible change of the nickel oxidation degree [7,8]. The *cisoid* conformation with the dicarbollide ligands turned by ~36 degree relative each other rotation angle is most favorable for the nickel(IV) bis(dicarbollide) complex, whereas the *transoid* conformation with ligand rotation angle 180 degree is preferred for nickel(III) bis(dicarbollide) [7,8,9,10]. Somewhat later, several such molecular switches were synthesized [11,12]; however, it was found that the difference in energies between the *transoid* and *gauche* rotamers in the synthesized nickel(III) complexes are negligibly low (~0.1 kcal/mol) [11]. In addition, it was found that nickel(IV) bis(dicarbollide) complexes are unstable in a number of solvents and are easily reduced to the corresponding nickel(III) complexes [13,14]. Thus, nickel bis(dicarbollide) complexes cannot be used to create effective electrochemically controlled molecular switches.

An alternative option involves the use of transition metal bis(dicarbollide) derivatives as structural elements in design of the complexation-driven molecular switches [15]. Additional stabilization of certain rotamers can be achieved by an introduction of appropriate substituents in the upper belt of the dicarbollide ligand, which results in the appearance of stabilizing interactions between the ligands. For instance, the introduction of iodine atoms to positions 8 and 8′ of the cobalt bis(dicarbollide) anion increases the energy difference between the *transoid* and *gauche* and the *transoid* and *cisoid* rotamers of [8,8′-I_2_-3,3′-Co(1,2-C_2_B_9_H_10_)_2_]^−^ in comparison with the parent anion from 0.4 to 4.7 kcal/mol and from 2.5 to 15.7 kcal/mol, respectively, due to intramolecular C–H···I–B hydrogen bonding between the ligands [16,17]. The bistability of molecular switches can be achieved by using substituents that are capable to form weak intramolecular hydrogen bonds between the dicarbollide ligands in the *transoid* conformation and stronger dative bonds with external transition metals in the *cisoid* conformation. Indeed, we found that the methylsulfanyl groups are able to stabilize the *transoid* conformation of the corresponding derivatives of cobalt and iron bis(dicarbollides) [8,8′-(MeS)_2_-3,3′-M(1,2-C_2_B_9_H_10_)_2_]^−^ (M = Co, Fe) due to formation of intramolecular C–H···S(Me)B hydrogen bonds [18,19]. On the other hand, in the presence of external transition metals or labile metal complexes, the methylsulfanyl groups form complexes with them, resulting in transformation of the *transoid* conformation of cobalt bis(dicarbollide) to the *cisoid* one [20,21].

In this contribution, we describe the synthesis and structure of the methylsulfanyl derivatives of nickel bis(dicarbollide).

## 2. Results and Discussion

### 2.1. Synthesis

**Scheme 1:**
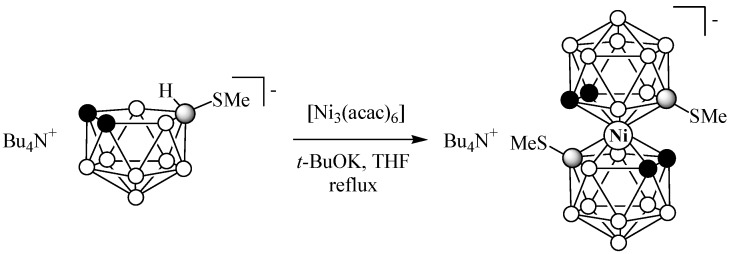
Synthesis of [8,8′-(MeS)_2_-3,3′-Ni(1,2-C_2_B_9_H_10_)_2_]^−^.

Our attempts to prepare 8,8′-di(methylsulfanyl) derivative of nickel(III) bis(dicarbollide) [8,8′-(MeS)_2_-3,3′-Ni(1,2-C_2_B_9_H_10_)_2_]^−^ in aqueous solution [22,23] by the reaction of NiCl_2_•6H_2_O with [10-MeS-7,8-C_2_B_9_H_11_]^−^ in 40% aqueous NaOH, as well as in nonaqueous media, using anhydrous NiCl_2_ in tetrahydrofuran [13,22], were unsuccessful. Nevertheless, we were able to synthesize the goal product by using the same approach that was used previously for the synthesis of 6,6’-substituted derivatives of nickel bis(dicarbollide) [11,12,24], namely the reaction of nickel(II) acetylacetonate complex [Ni_3_(acac)_6_] with (Bu_4_N)[10-MeS-7,8-C_2_B_9_H_11_] in tetrahydrofuran in the presence of *t-*BuOK (Scheme 1).

**Scheme 2:**
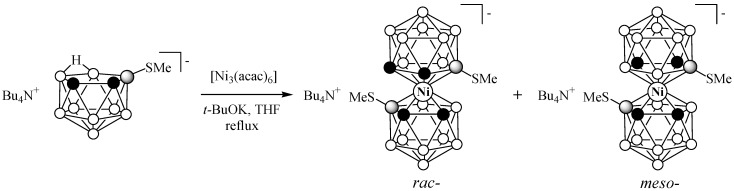
Synthesis of [4,4′-(MeS)_2_-3,3′-Ni(1,2-C_2_B_9_H_10_)_2_]^−^ and [4,7′-(MeS)_2_-3,3′-Ni(1,2-C_2_B_9_H_10_)_2_]^−^.

The similar reaction of [Ni_3_(acac)_6_] with unsymmetrically substituted methylsulfanyl derivative of *nido*-carborane (Bu_4_N)[9-MeS-7,8-C_2_B_9_H_11_] results in the mixture of *rac*-(Bu_4_N)[4,4′-(MeS)_2_-3,3′- Ni(1,2-C_2_B_9_H_10_)_2_] and *meso*-(Bu_4_N)[4,7′-(MeS)_2_-3,3′-Ni(1,2-C_2_B_9_H_10_)_2_] (Scheme 2).

Separation of the *rac-* and *meso-*isomers of related anionic nickel(III) complex [1,1′(2′)-Me_2_-3,3′-Ni(1,2-C_2_B_9_H_10_)_2_]^−^ by their oxidation to the corresponding neutral nickel(IV) complexes has been described in the literature [25]. However, it was found that attempts to oxidize both diastereomeric (Bu_4_N)[4,4′(7′)-(MeS)_2_-3,3′-Ni(1,2-C_2_B_9_H_10_)_2_] and symmetrically substituted (Bu_4_N)[8,8′-(MeS)_2_-3,3′-Ni(1,2-C_2_B_9_H_10_)_2_] nickel(III) complexes to the corresponding nickel(IV) complexes with iron chloride in acetonitrile lead to their decomposition. Therefore, by analogy with the corresponding derivatives of cobalt bis(dicarbollide) [18], column chromatography was used to separate the nickel(III) diastereomeric complexes. The ^11^B NMR spectra of all the obtained paramagnetic complexes (Figures S1–S3) are quite characteristic; however, unlike the similar derivatives of paramagnetic iron bis(dicarbollide) [19,26,27], their interpretation is not possible. Therefore, an X-ray diffraction study is crucial to determine the structure of the diastereomers.

### 2.2. X-Ray Diffraction Study

Since the chemistry of nickel bis(dicarbollides) is much less studied than the chemistry of its iron and cobalt analogues, only a limited number of X-ray structures of its derivatives are known. Like the parent nickel bis(dicarbollide), its derivatives contain substituents in the lower belt of the dicarbollide ligand adopt *transoid* conformation in the nickel(III) complexes and *cisoid* conformation in the nickel(IV) complexes [8,11,23]. In the case of derivatives containing substituents in the upper belt of the ligand, the structure of the complexes depends not only on the oxidation state of the metal, but also on the type and position of the substituents. The *transoid* conformation was found both in 1,1′-Me_2_- and 1,2′-Me_2_-derivatives of nickel(IV) bis(dicarbollide), whereas the corresponding nickel(III) complexes adopt *gauche* and *transoid* conformations, respectively [25]. The *gauche* conformation stabilized by two intramolecular C–H···OMe hydrogen bonds was found in the 8,8′-(MeO)_2_-derivative of nickel(IV) bis(dicarbollide) [28]. The same conformation stabilized by two intramolecular C–H···SMe_2_ bonds was found also in the 8,8′-(Me_2_S)_2_-derivative of nickel(II) bis(dicarbollide) [29]. The latter examples demonstrate the role of substituents capable of forming intramolecular hydrogen bonds in the stabilization of rotamers, which are generally less preferred in the absence of such substituents.

**Figure 1:**
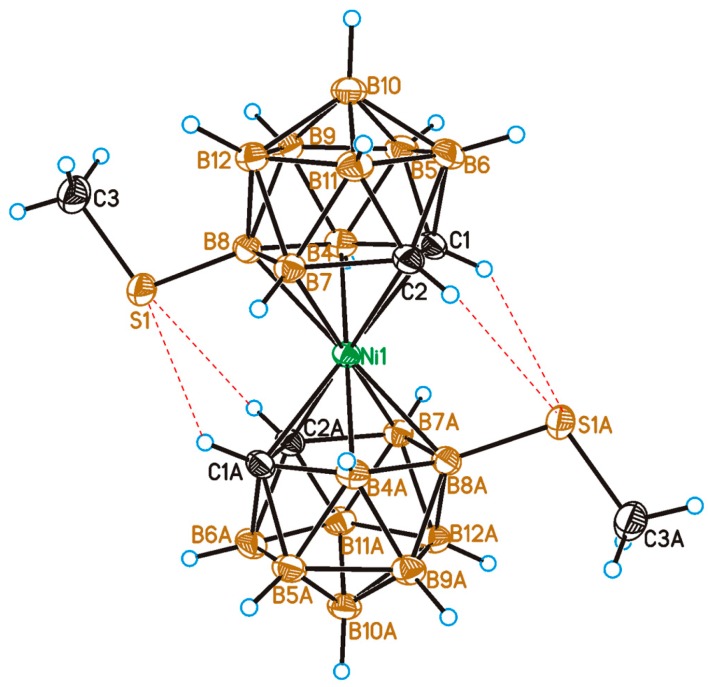
Thermal ellipsoid plot (50%) and labeling scheme for [8,8′-(MeS)_2_-3,3′-Ni(1,2-C_2_B_9_H_10_)_2_]^−^.

Single-crystal X-ray diffraction study of (Bu_4_N)[8,8′-(MeS)_2_-3,3′-Ni(1,2-C_2_B_9_H_10_)_2_] demonstrated that, similarly to the corresponding derivatives of iron(II) and cobalt(III) bis(dicarbollides) [18,19], the dicarbollide ligands in the symmetrically substituted anion [8,8′-(MeS)_2_-3,3′-Ni(1,2-C_2_B_9_H_10_)_2_]^−^ adopt *transoid* configuration stabilized by four (two pairs) intramolecular CH···S contacts (2.78–2.83 Å) between the dicarbollide ligands (Figure 1).

**Figure 2:**
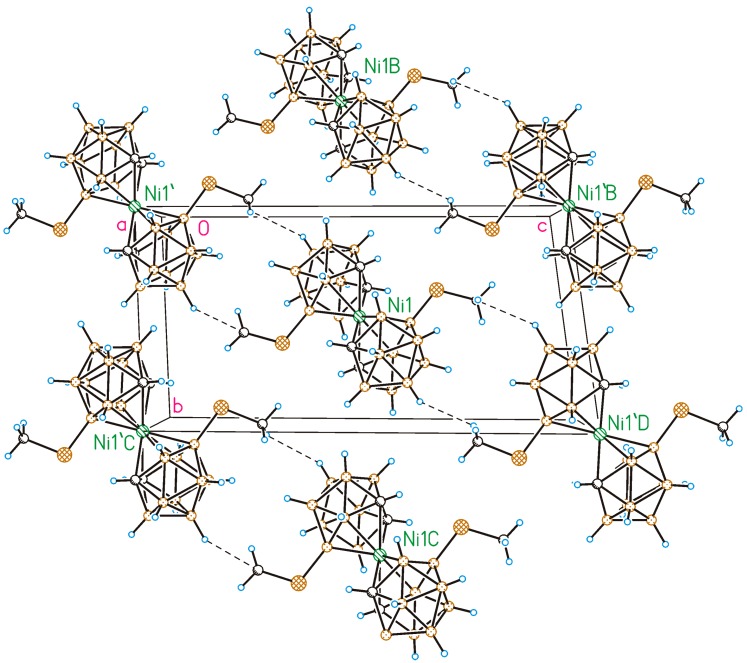
The structure of anionic layer. The shortest H···H contacts (BH(10)···H_3_C of 2.58 Å) are observed between A and A’ anions. Each anion is surrounded by only two anions of the same type and by four anions of the different type.

An asymmetric unit cell contains three structural units: one cation and two halves of anion (A and A’). The crystal structure is formed by alternate layers parallel to the *bc* crystallographic plane (Figure S4). Most of intermolecular interactions between different structural units are of van-der-Waals-type. However, it is interesting to note that, in anionic layers, interactions between A and A’ are somewhat more pronounced (Figure 2), and that might be the reason of formation of two independent anions. Such conclusion can be supported by many literature examples in which interactions between different structural units appear to be stronger [30,31,32].

**Figure 3:**
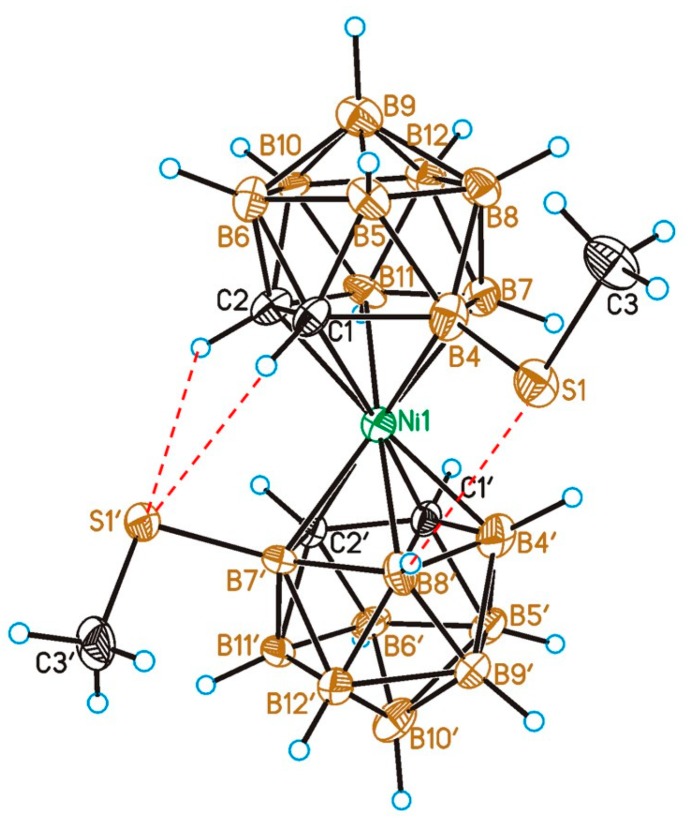
Thermal ellipsoid plot (50%) and labeling scheme for [4,7′-(MeS)_2_-3,3′-Ni(1,2-C_2_B_9_H_10_)_2_]^−^.

Single-crystal X-ray diffraction study of the tetrabutylammonium salt of the *meso*-diastereomer (Bu_4_N)[4,7′-(MeS)_2_-3,3′-Ni(1,2-C_2_B_9_H_10_)_2_] revealed that it is isostructural with the corresponding iron [19] and cobalt [18] complexes. Similarly to the corresponding derivatives of iron(III) and cobalt(III) bis(dicarbollides), the dicarbollide ligands in the [4,7′-(MeS)_2_-3,3′-Ni(1,2-C_2_B_9_H_10_)_2_]^−^ anion adopt *gauche* configuration stabilized by two CH···S and one BH···S intramolecular contacts (Figure 3).

**Figure 4:**
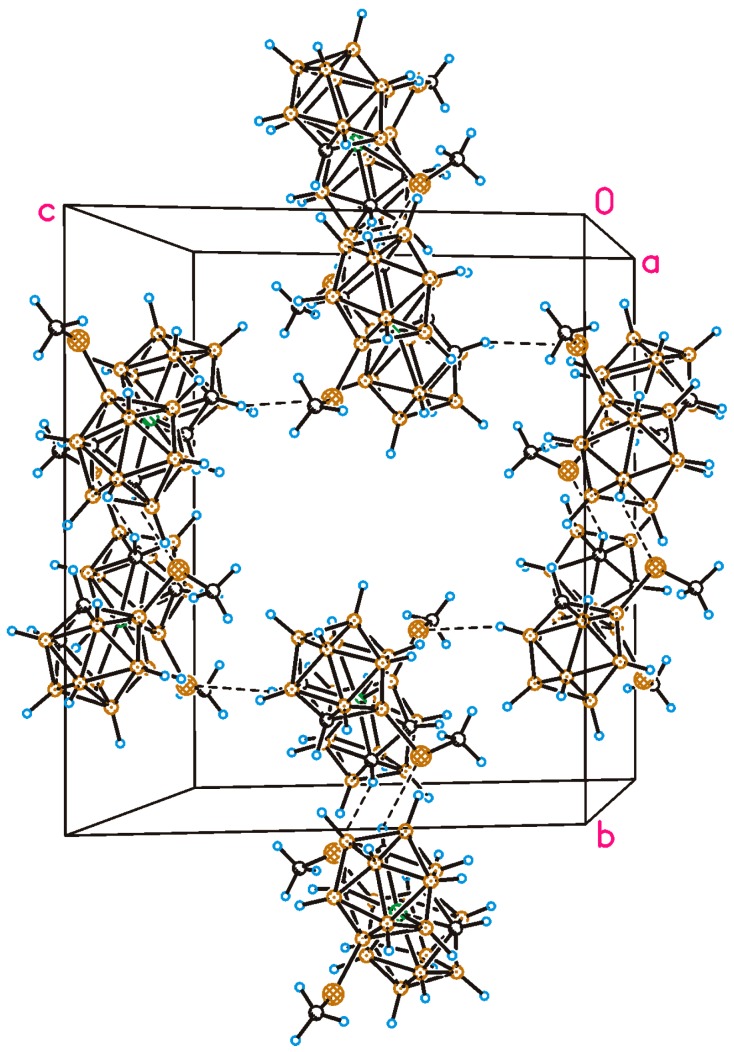
The structure of anionic layer stabilized by weak C1’–H1···S1 (C–H is 1.12 Å, C···S is 3.944(12) Å, H···S is 2.89 Å, <CHS is 158°) and C2–H2···S1’ (C–H is 1.12 Å, C···S is 3.732(13) Å, H···S is 2.77 Å, <CHS is 144°) hydrogen bonds.

An asymmetric unit cell consists of one cation and one anion. Crystal packing of (Bu_4_N)[4,7′-(MeS)_2_-3,3′-Ni(1,2-C_2_B_9_H_10_)_2_] is also built up of alternate cationic and anionic layers (Figure S5). However, in contrast to (Bu_4_N)[8,8′-(MeS)_2_-3,3′-Ni(1,2-C_2_B_9_H_10_)_2_], layers in (Bu_4_N)[4,7′-(MeS)_2_-3,3′-Ni(1,2-C_2_B_9_H_10_)_2_] adopt corrugated shape, and cationic layers penetrate into anionic ones. Also, in contrast to (Bu_4_N)[8,8′-(MeS)_2_-3,3′-Ni(1,2-C_2_B_9_H_10_)_2_], stabilization of anionic layers in (Bu_4_N)[4,7′-(MeS)_2_-3,3′-Ni(1,2-C_2_B_9_H_10_)_2_] is provided by significant contribution of weak C_carb_–H···S hydrogen bonds (Figure 4).

Despite that 4,7′-bis(methylsulfanyl) derivatives of iron, cobalt, and nickel bis(dicarbollides) are isostructural, a thorough analysis of the structural features of these complexes (Table 1) revealed that, despite the largest size of the nickel cation [33] that clearly reflects at the metal···dicarbollide ligand distance, the BH···S chalcogen bond in the nickel complex is much shorter (2.53 Å) than in other complexes (2.61 and 2.70 Å for the iron and cobalt complexes, respectively) and probably plays a larger role in stabilization of the *gauche* conformation.

Generally, the formation of a chalcogen bond is explained in terms of the positive electrostatic potential present on the outermost portion of the chalcogen’s surface, opposite to the R–Ch bonds. This region of positive electrostatic potential, which is called the “σ-hole”, can interact attractively with negative sites of the same molecule or other molecules. Due to the presence of lone pairs of the chalcogen atom, a σ-hole is usually surrounded by a region of negative molecular electrostatic potential, and this determines a high directionality of the chalcogen bonds [34,35,36]. The acceptors of the chalcogen bond may be atoms with an unshared electron pair (e.g., pyridine or amine nitrogen atom, and ether or carbonyl group oxygen atom), π-system (e.g., double or triple bond and arene moiety), or anion (e.g., halide anion and polyatomic oxyanion) [37]. To the best of our knowledge, there is only one reference in the literature to boron hydrides as acceptors of chalcogen bonds [38], and examples of chalcogen bonds with other hydrides are quite rare, as well [39,40].

Therefore, in order to assess the contribution of various factors to the stabilization of the *gauche* conformation of the *meso*-diastereomer [4,7′-(MeS)_2_-3,3′-Ni(1,2-C_2_B_9_H_10_)_2_]^−^, as well as to obtain information on the structure of the *rac*-diastereomer [4,4′-(MeS)_2_-3,3′-Ni(1,2-C_2_B_9_H_10_)_2_]^−^, we carried out quantum chemical calculations of the all synthesized complexes.

### 2.3. Quantum Chemical Calculations

The parent nickel(III) bis(dicarbollide) [3,3′-Ni(1,2-C_2_B_9_H_11_)_2_]^−^ is known to prefer the *transoid* configuration, while the *gauche* form is only slightly less (~1 kcal/mol) preferred [7,41]. Our DFT calculations using the BP86 functional with cc-pvdz basis set gave the similar result with the *transoid* configuration preferred over the *gauche* one by only 0.4 kcal/mol, whereas the *cisoid*-rotamer is energetically disfavored by as much as 4 kcal/mol. These allow us to suppose that weak noncovalent intramolecular interactions could switch preferability of the *transoid* and *gauche* rotamers.

**Figure 5:**
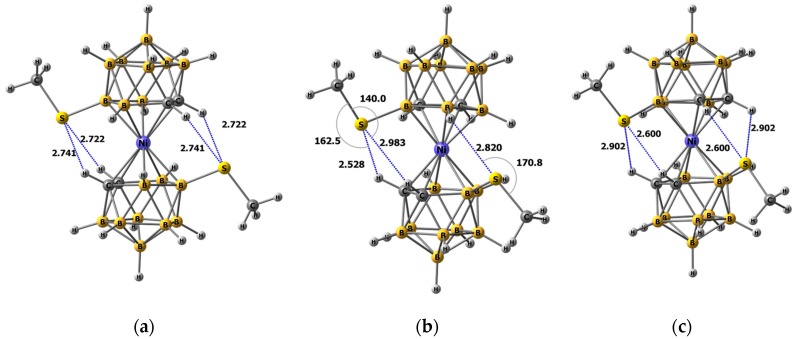
DFT-optimized structures of methylsulfanyl derivatives of nickel(III) bis(dicarbollide): (**a**) [8,8′-(MeS)_2_-3,3′-Ni(1,2-C_2_B_9_H_10_)_2_]^−^ isomer; (**b**) [4,7′-(MeS)_2_-3,3′-Ni(1,2-C_2_B_9_H_10_)_2_]^−^ isomer; and (**c**) [4,4′-(MeS)_2_-3,3′-Ni(1,2-C_2_B_9_H_10_)_2_]^−^ isomer.

The *transoid* rotamer of the [8,8′-(MeS)_2_-3,3′-Ni(1,2-C_2_B_9_H_10_)_2_]^−^ isomer featuring four stabilizing CH···S hydrogen bonds (r(H···S) = 2.72–2.74 Å, E_BCP_ = −1.8 ÷ −1.6 kcal/mol, Figure 5) become unambiguously global minima preferred over the *gauche* rotamer by 3.7/4.0 kcal/mol (ΔE/ΔG_298_ terms). The *gauche* rotamer is skewed to the 117.4° from the standard value of 108°, in order to provide shorter and stronger CH···S bonds (r(H···S) = 2.66 Å, E_BCP_ = 2.0 kcal/mol), but only two such bonds could form. The *cisoid* rotamer has only S…S stabilizing interaction, and this pushes it as high as ΔE = +10.3 kcal/mol relative to the *transoid* one.

For the [4,7′-(MeS)_2_-3,3′-Ni(1,2-C_2_B_9_H_10_)_2_]^−^ isomer, the *transoid* rotamer has two equal BH···S chalcogen bonds (r(H···S) = 2.56 Å, angle C^Me^S···H = 170.7°, E_BCP_ = −2.5 kcal/mol. Despite the possibility of the CH···S interactions in this rotamer, such a configuration was not found as a minimum. The *gauche* rotamer was found to be slightly preferred over the *transoid* one (ΔE = 0.4; ΔG_298_ = 0.8 kcal/mol), and exactly this was revealed in the crystal. This rotamer is stabilized by two nonequivalent CH···S interactions (r(H···S) = 2.53 and 2.98 Å, E_BCP_ = −2.6 and −1.1 kcal/mol) and one BH···S chalcogen bond (r(H···S) = 2.82 Å, angle C^Me^S···H = 170.8°, and E_BCP_ = −1.4 kcal/mol, Figure 5). The *cisoid* rotamer has S···S (r(S···S) = 3.53 Å, E_BCP_ = −1.2 kcal/mol), CH···S (r(H···S) = 2.60 Å, and E_BCP_ = −3.0 kcal/mol), and BH···S (r(H···S) = 2.89 Å and E_BCP_ = −1.5 kcal/mol) interactions, but they are not enough to shift the energetic preference, leaving the *cisoid* rotamer to be ΔE = +4.3 kcal/mol higher than the *gauche* one.

For the [4,4′-(MeS)_2_-3,3′-Ni(1,2-C_2_B_9_H_10_)_2_]^−^ isomer, the *transoid* rotamer should have two sulfur atoms close to each other. Despite the possibility of stabilizing CH···S interactions in this rotamer sulfur atoms repulsion leads to the mutual rotation of the dicarbollide ligands, pushing molecules to the *gauche* configuration. Thus, no *transoid* rotamer was found on the PES, but two *gauche* and two *cisoid* rotamers were found (due to molecular symmetry). At that, the most favored one is a *gauche1* rotamer, which features four CH···S interactions (r(H···S) = 2.60–2.90 Å, E_BCP_ = −2.2 ÷ −1.3 kcal/mol, Figure 5). The another *gauche2* rotamer is unfavored by ΔE = +5.4 kcal/mol due to only two BH···S and S···S stabilizing interactions. Both the *cisoid* rotamers remain highly unfavorable (ΔE = +8.2 and +6.0 kcal/mol), despite the presence of four BH···S (*cisoid1*) and two BH···S with two CH···S (*cisoid2*) interactions. At that, the *cisoid2* rotamer featuring CH···S interactions is slightly preferred.

In general, since CH···S hydrogen bonds are formed with lone pairs of sulfur atoms, the formation of two such interactions per sulfur atom in methylsulfanyl derivatives is allowed. This results in stabilization of *gauche1*-[4,4′-(MeS)_2_-3,3′-Ni(1,2-C_2_B_9_H_10_)_2_]^−^ and *transoid*-[8,8′-(MeS)_2_-3,3′- Ni(1,2-C_2_B_9_H_10_)_2_]^−^ rotamers. In contrast, only one intramolecular chalcogen bond can be effectively formed, since only one σ-hole in the extension of the CH_3_-S bond is pointed in the appropriate direction. The formation of the chalcogen bond with hydride ligand requires rotation of the SMe group to form a linear fragment of C–S···H–B, which, in turn, leads to disruption of the CH^Me^···HB interactions. As a result, the *transoid* rotamer of the 4,7′ isomer with strongest chalcogen bonds have only two interactions of the Me group with BH (one per the Me group). Notable that the least favored among all conformers *cisoid*-rotamer of the 4,4′-isomer featuring four S···HB interactions has no interactions of the Me groups with the boron cage at all. Therefore, with a small difference in energy between *gauche* and *transoid* rotamers, the possibility of formation of four stabilizing CH···S interactions unambiguously leads to formation of the corresponding rotamer. Such a conformer is further stabilized due to a larger amount of CH^Me^···HB interactions. When the atomic configuration does not allow such interactions, chalcogen bonds affect the rotamer stabilization.

## 3. Materials and Methods 

### 3.1. General Considerations

All reactions were performed in an inert nitrogen atmosphere while using standard Schlenk techniques. (Bu_4_N)[10-MeS-7,8-C_2_B_9_H_11_], (Bu_4_N)[9-MeS-7,8-C_2_B_9_H_11_], and [Ni(acac)_2_]_3_ were prepared as described in the literature [42,43,44]. Anhydrous tetrahydrofuran was prepared by using the standard procedure [45]. Acros Organics silica gel (0.060–0.200 mm) was used for column chromatography. Thin-layer chromatograms (DC Kieselgel F_254_ silica gel on aluminum plates, Merck) were visualized, using 0.1% PdCl_2_ in 3 M of HCl(aq). The ^11^B NMR spectra at 128.4 MHz were recorded with a Bruker Avance-400 spectrometer (Bruker Biospin AG, Fällanden, Switzerland) and referenced by using BF_3_∙Et_2_O as the external standard. High-resolution mass spectra (HRMS) were measured on a Bruker micrOTOF II instrument (Bruker Daltonics, Bremen, Germany), using electrospray ionization (ESI). The measurements were done in a negative ion mode (3200 V); mass range from m/z 50 to m/z 3000; external or internal calibration was done with ESI Tuning Mix, Agilent (Santa Clara, CA, USA). A syringe injection was used for solutions in acetonitrile (flow rate 3 mL/min). Nitrogen was applied as a dry gas; the interface temperature was set at 180 °C.

### 3.2. Synthesis of Methylsulfanyl Derivatives of Nickel(III) Bis(Dicarbollide)

#### 3.2.1. Synthesis of (Bu_4_N)[8,8′-(MeS)_2_-3,3′-Ni(1,2-C_2_B_9_H_10_)_2_]

First, 0.25 g (2.20 mmol) of ^t^BuOK was added to solution of 0.41 g (1.11 mmol) of (Bu_4_N)[10-MeS-7,8-C_2_B_9_H_11_] in 40 mL of THF and heated under reflux for 40 min. Then, 0.21 g (0.28 mmol) of [Ni(acac)_2_]_3_ was added, and the reaction mixture was heated under reflux for 24 h. The reaction mixture was allowed to cool to room temperature and concentrated to dryness in vacuo. The residue was treated with 50 mL of dichloromethane and 50 mL of water. Organic layer was separated, dried over Na_2_SO_4_, filtered, and evaporated in vacuo. The crude product was purified by column chromatography on silica, using dichloromethane as eluent to obtain 0.20 g (55%) of brown product. ^11^B NMR (acetone-*d*_6_, ppm): δ = 19.6, 9.8, −119.8. HRMS (ESI): calcd. for C_6_B_18_H_26_NiS_2_ [M]^−^ 415.2630; found 415.2621

#### 3.2.2. Synthesis of (Bu_4_N)[4,4′-(MeS)_2_-3,3′-Ni(1,2-C_2_B_9_H_10_)_2_] (rac-isomer) and (Bu_4_N)[4,7′-(MeS)_2_-3,3′- Ni(1,2-C_2_B_9_H_10_)_2_] (meso-isomer)

First, 0.59 g (5.21 mmol) of ^t^BuOK was added to a solution of 1.10 g (2.61 mmol) of (Bu_4_N)[9-MeS-7,8-C_2_B_9_H_11_] in 40 mL of THF and heated under reflux for 40 min. Then, 0.50 g (0.65 mmol) of [Ni(acac)_2_]_3_ was added, and the reaction mixture was heated under reflux for 24 h. The reaction mixture was allowed to cool to room temperature and concentrated to dryness in vacuo. The residue was treated with 50 mL of dichloromethane and 50 mL of water. The organic layer was separated, washed over Na_2_SO_4_, filtered, and evaporated in vacuo. The mixture of isomers was separated by column chromatography on silica with the mixture of dichloromethane and chloroform (4:1, v/v) as eluent to obtain 0.26 g (30%, R_f_ = 0.75) of *rac-*isomer and 0.21 g (24%, R_f_ = 0.50) of *meso*-isomer as brown solids. *Rac*-isomer: ^11^B NMR (acetone-*d*_6_, ppm): δ = 69.8, 6.3, −1.4, −38.9, −74.3. HRMS (ESI): calcd. for C_6_B_18_H_26_NiS_2_ [M]^−^ 415.2630; found 415.2625. *meso*-isomer: ^11^B NMR (acetone-*d*_6_, ppm): δ = 52.2, 6.8, −26.7, −37.8, −42.0. HRMS (ESI): calcd. for C_6_B_18_H_26_NiS_2_ [M]^−^ 415.2630; found 415.2624.

### 3.3. X-Ray Diffraction Study

X-ray experiments for compounds (Bu_4_N)[8,8′-(MeS)_2_-3,3′-Ni(1,2-C_2_B_9_H_10_)_2_] and (Bu_4_N)[4,7′- (MeS)_2_-3,3′-Ni(1,2-C_2_B_9_H_10_)_2_] were carried out by using SMART APEX2 CCD diffractometer (λ(Mo-Kα) = 0.71073 Å, graphite monochromator, ω-scans) at 120 K. Collected data were processed by the SAINT and SADABS programs incorporated into the APEX2 program package [46]. The structures were solved by the direct methods and refined by the full-matrix least-squares procedure against F2 in anisotropic approximation. The refinement was carried out with the SHELXTL program [47]. In the case of (Bu_4_N)[8,8′-(MeS)_2_-3,3′-Ni(1,2-C_2_B_9_H_10_)_2_], all carborane hydrogen atoms were refined isotropically, without constraints. In contrast, reflection ability of (Bu_4_N)[8,8′-(MeS)_2_-3,3′-Ni(1,2- C_2_B_9_H_10_)_2_] was extremely weak; therefore, all hydrogen atoms were refined within the riding model. The details of data collection and crystal structures refinement are summarized in Table S1. CCDC numbers (No.1966075 for (Bu_4_N)[8,8′-(MeS)_2_-3,3′-Ni(1,2-C_2_B_9_H_10_)_2_] and No.1966074 for (Bu_4_N)[4,7′- (MeS)_2_-3,3′-Ni(1,2-C_2_B_9_H_10_)_2_]) contain the supplementary crystallographic data for this paper. These data can be obtained free of charge via www.ccdc.cam.ac.uk/data_request/cif.

### 3.4. Quantum Chemical Calculations

The geometry optimizations were performed with the BP86 functional [48,49] with cc-pvdz basis set [50,51,52,53], without any symmetry restrictions, using Gaussain09 [54]. The tight SCF convergence criteria and ultrafine grid were applied. All minima on the PES were confirmed to have no negative values in their diagonalized force constants matrix. The QTAIM analysis was performed by using the *AIMALL* [55] and *MULTIWFN* [56] programs package based on the wave function obtained by the BP86 calculations. The energies of noncovalent intramolecular interactions were calculated by using the correlation between the interaction energy (E_BCP_) and the value of the potential energy density function V_C_(r) at the corresponding critical point (3, −1): E_BCP_ = 0.5∙V_C_(r) (Table S2) [57,58]. Such approach is widely used for energetic analysis of intermolecular interactions of different types [42,43,59,60].

## 4. Conclusions

Symmetrically and unsymmetrically substituted methylsulfanyl derivatives of nickel(III) bis(dicarbollide) (Bu_4_N)[8,8′-(MeS)_2_-3,3′-Ni(1,2-C_2_B_9_H_10_)_2_], (Bu_4_N)[4,4′-(MeS)_2_-3,3′-Ni(1,2-C_2_B_9_H_10_)_2_], and (Bu_4_N)[4,7′-(MeS)_2_-3,3′-Ni(1,2-C_2_B_9_H_10_)_2_] were synthesized, starting from [Ni(acac)_2_]_3_ and the corresponding methylsulfanyl derivatives of *nido*-carborane (Bu_4_N)[10-MeS-7,8-C_2_B_9_H_11_] and (Bu_4_N)[10-MeS-7,8-C_2_B_9_H_11_]. Structures of the synthesized metallacarboranes were studied by single-crystal X-ray diffraction and quantum chemical calculations. The symmetrically substituted 8,8′-isomer was found to adopt *transoid* conformation stabilized by two pairs of intramolecular C–H···S hydrogen bonds between the dicarbollide ligands. The unsymmetrically substituted 4,7′-isomer adopts *gauche* conformation, which is stabilized by two nonequivalent C–H···S hydrogen bonds and one short chalcogen B–H···S bond (2.53 Å, −1.4 kcal/mol). The *gauche* conformation stabilized by two pairs of C–H···S hydrogen bonds was found to be also preferred for the 4,7′-isomer. At a small difference in energy between the *gauche* and *transoid* rotamers, the possibility of formation of four stabilizing CH···S interactions unambiguously leads to the formation of the corresponding rotamers. When the atomic configuration does not allow such interactions, the formation of BH···S chalcogen bonds affects the rotamer stabilization.

## Figures and Tables

**Table 1:** Some X-ray structure parameters of [4,7′-(MeS)_2_-3,3′-M(1,2-C_2_B_9_H_10_)_2_]^−^ (M = Fe, Co, Ni) anions.

	M = Fe	M = Co	M = Ni
C(1)H(1)···S(1′) distance, Å	2.75	2.71(3)	2.68
C(2)H(2)···S(1′) distance, Å	2.69	2.70(3)	2.83
S(1′)Me group rotation angle, º	27.9(2)	29.59(14)	30.1(9)
M···ligand plane distance, Å	1.530(3)	1.474(2)	1.555(10)
B(8′)H(8′)···S(1) distance, Å	2.61	2.70(3)	2.53
S(1)Me group rotation angle, º	6.5(2)	6.66(13)	10.9(9)
M···ligand plane distance, Å	1.538(2)	1.482(2)	1.582(9)
ligand rotation angle, º	102.4(3)	104.2(2)	105.0(11)

## Supplementary Materials

The following are available online at https://www.mdpi.com/1420-3049/24/24/4449/s1. Figure S1: ^11^B NMR spectrum of (Bu_4_N)[8,8’-(MeS)_2_-Ni(C_2_B_9_H_10_)_2_]. Figure S2: ^11^B NMR spectrum of (Bu_4_N)[4,4’-(MeS)_2_- Ni(C_2_B_9_H_10_)_2_]. Figure S3: ^11^B NMR spectrum of (Bu_4_N)[4,7’-(MeS)_2_-Ni(C_2_B_9_H_10_)_2_]. Figure S4: Crystal packing fragment of (Bu_4_N)[8,8′-(MeS)_2_-3,3′-Ni(1,2-C_2_B_9_H_10_)_2_]. Figure S5: Crystal packing fragment of (Bu_4_N)[4,7′- (MeS)_2_-3,3′-Ni(1,2-C_2_B_9_H_10_)_2_]. Table S1: Crystallographic data for compounds (Bu_4_N)[8,8′-(MeS)_2_-3,3′- Ni(1,2-C_2_B_9_H_10_)_2_] and (Bu_4_N)[4,7′-(MeS)_2_-3,3′-Ni(1,2-C_2_B_9_H_10_)_2_]. Table S2: Characteristics of (3,-1) bond critical points associated with the intramolecular interactions of SMe group title.
