# Supplementary material for: Synthesis and Structure of Methylsulfanyl Derivatives of Nickel Bis(Dicarbollide)
Source: Molecules. 2019 Dec 4;24(24):4449. doi: 10.3390/molecules24244449 (PMC6943677; doi:10.3390/molecules24244449)
Supplement: Supplementary file 1 [file molecules-24-04449-s001.pdf]

Supplementary Materials

Synthesis and Structure of Methylsulfanyl Derivatives of Nickel Bis(Dicarbollide)

Sergey A. Anufriev [1], Kyrill Yu. Suponitsky [1], Oleg A. Filippov [1,2], Igor B. Sivaev [1,3,*]

[1] A.N. Nesmeyanov Institute of Organoelement Compounds, Russian Academy of Sciences, 28 Vavilov Str, Moscow, 119991, Russia; trueman476@mail.ru (S.A.A.), kirshik@yahoo.com (K.Y.S.), h-bond@ineos.ac.ru (O.A.F.)
[2] Shemyakin-Ovchinnikov Institute of Bioorganic Chemistry, Russian Academy of Sciences, 16/10 Miklukho-Maklay Str., Moscow 117997, Russia
[3] Basic Department of Chemistry of Innovative Materials and Technologies, G.V. Plekhanov Russian University of Economics, 36 Stremyannyi Line, 117997 Moscow 117997, Russia
* Correspondence: sivaev@ineos.ac.ru; Tel.: +7-916-590-2025

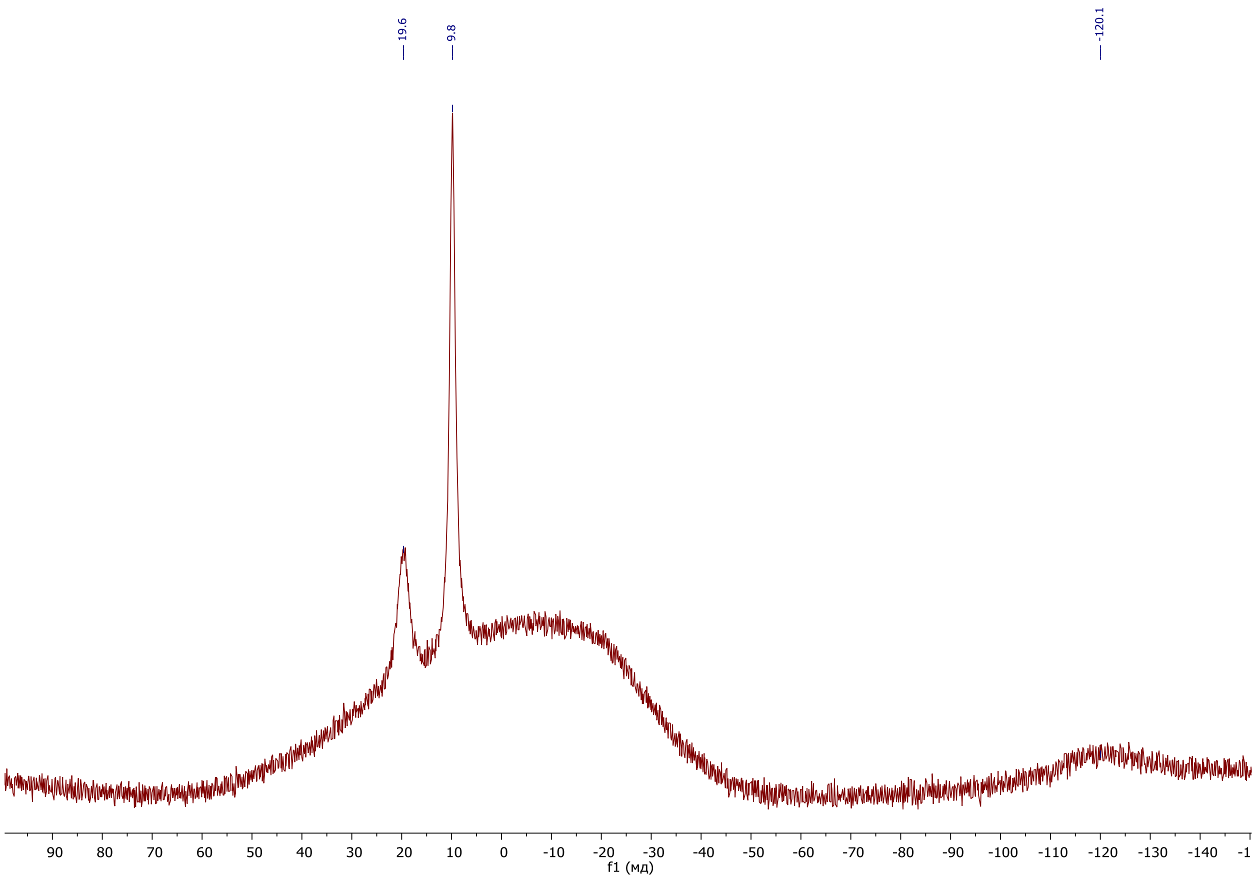

Figure S1. ^{11}B NMR spectrum of $(Bu_4N)[8,8`-(MeS)_2-Ni(C_2B_9H_{10})_2]$ in acetone-d_6.

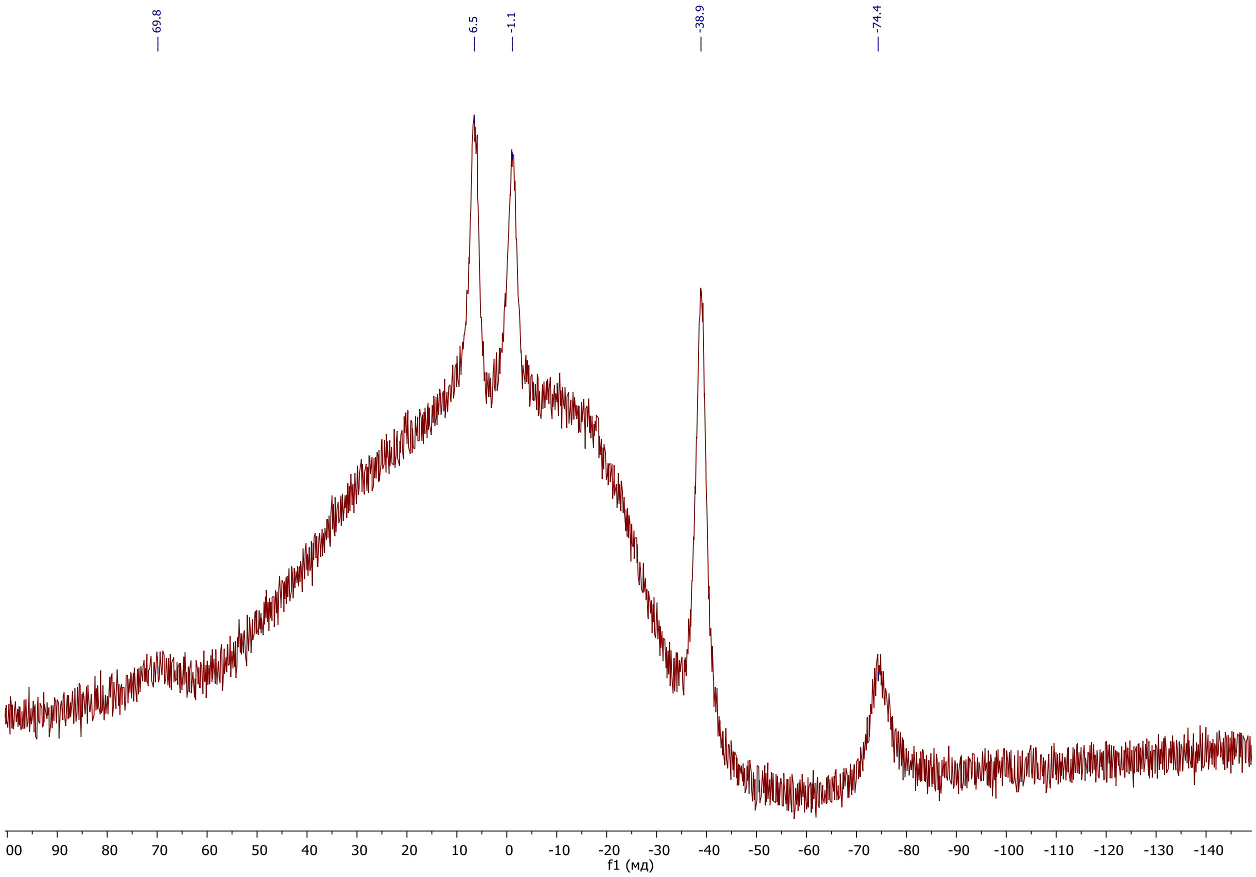

Figure S2. ^{11}B NMR spectrum of $(Bu_4N)[4,4`-(MeS)_2-Ni(C_2B_9H_{10})_2]$ in acetone-d_6.

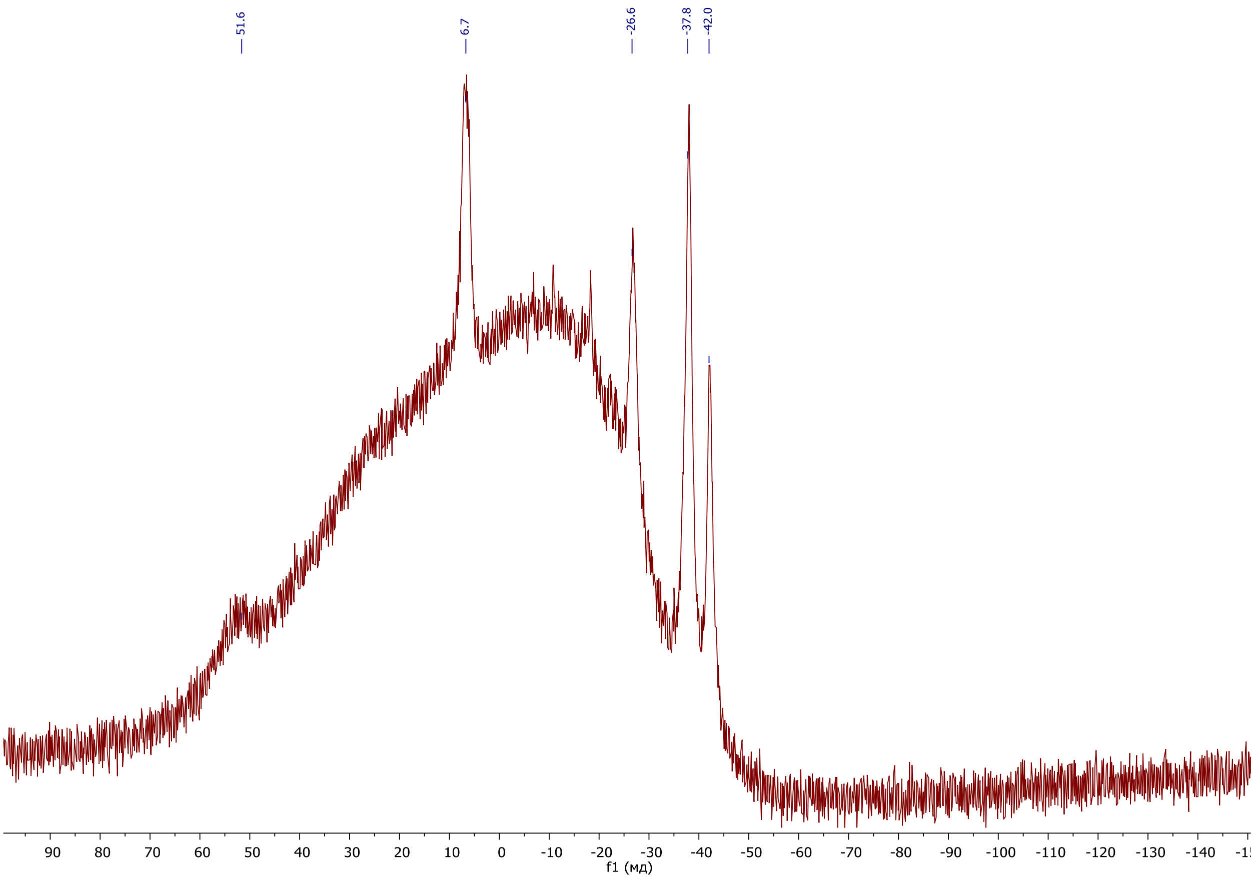

Figure S3. ^{11}B NMR spectrum of $(Bu_4N)[4,7`\text{-}(MeS)_2\text{-}Ni(C_2B_9H_{10})_2]$ in acetone-d_6.

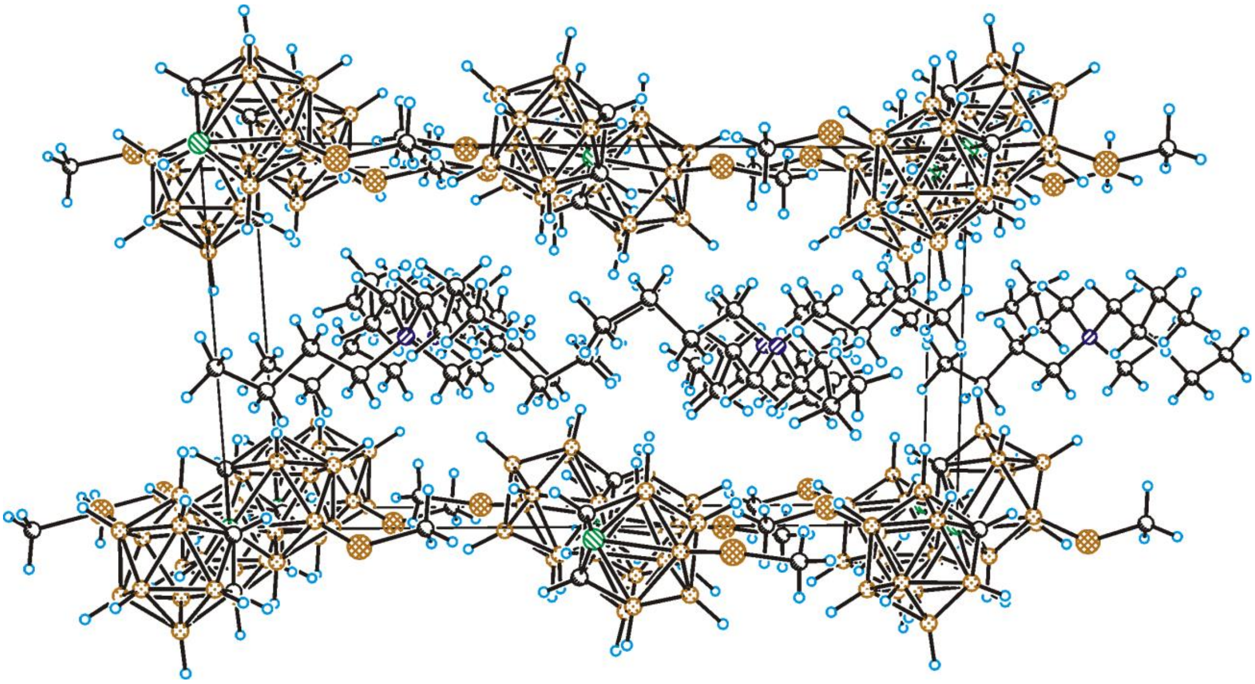

Figure S4. Crystal packing fragment of $(Bu_4N)[8,8'\text{-}(MeS)_2\text{-}3,3'\text{-}Ni(1,2\text{-}C_2B_9H_{10})_2]$. Alternation of anionic and cationic layers parallel to the *bc* crystallographic plane is shown.

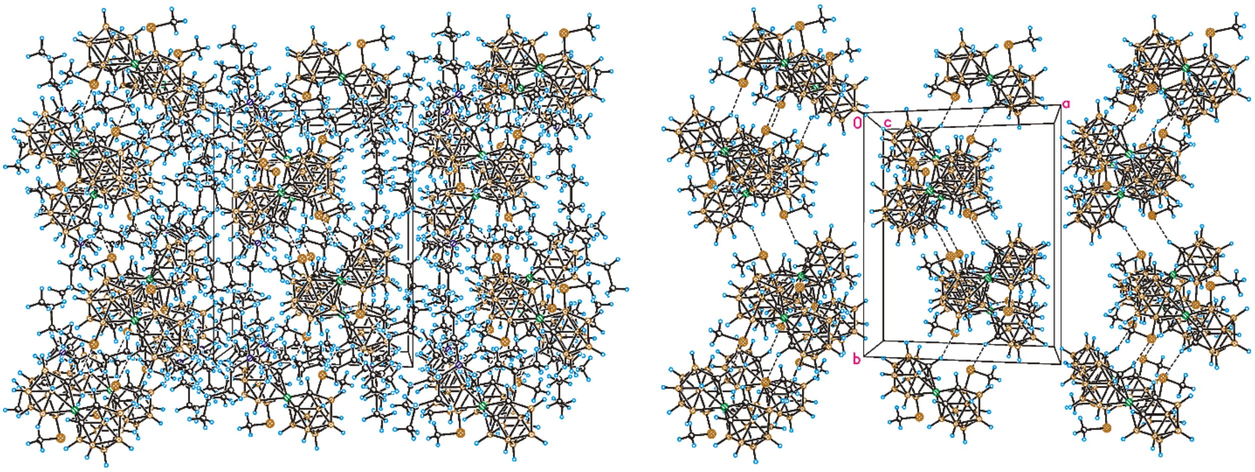

Figure S5. Crystal packing fragment of $(Bu_4N)[4,7'\text{-}(MeS)_2\text{-}3,3'\text{-}Ni(1,2\text{-}C_2B_9H_{10})_2]$. Alternation of anionic and cationic layers parallel to the *bc* crystallographic plane is shown. Total packing is presented on the left side while anion disposition is presented on the left side for more clear understanding. Cationic layers penetrate into anionic layers.

Table S1. Crystallographic data for compounds $(Bu_4N)[8,8'-(MeS)_2-3,3'-Ni(1,2-C_2B_9H_{10})_2]$(**1**) and $(Bu_4N)[4,7'-(MeS)_2-3,3'-Ni(1,2-C_2B_9H_{10})_2]$ (**2**).

	**1**	**2**
formula	$C_6H_{26}B_{18}NiS_2^-$, $C_{16}H_{36}N^+$	$C_6H_{26}B_{18}NiS_2^-$, $C_{16}H_{36}N^+$
fw	658.13	658.13
crystal system	Triclinic	Monoclinic
space group	*P*-1	$P2_1/c$
a, Å	10.4662(5)	15.004(4)
b, Å	10.4746(5)	17.500(5)
c, Å	18.6951(9)	14.919(4)
α, deg.	83.6560(10)	90.0
β, deg.	86.5750(10)	111.443(6)
γ, deg.	64.9800(10)	90.0
V, $Å^3$	1845.64(15)	3646.0(17)
Z	2	4
d_{cryst}, $g \cdot cm^{-3}$	1.184	1.199
F(000)	702	1404
μ, mm^{-1}	0.657	0.665
θ range, deg.	1.1 – 26.0	1.9 – 24.0
independent reflections / R_{int}	7270	5711
Completeness to theta θ, %	99.9	99.9
refined parameters	486	423
GOF (F^2)	1.072	1.033
reflections with $I>2\sigma(I)$	5668	2971
$R_1(F)$ ($I>2\sigma(I)$)[a]	0.0413	0.0761
$wR_2(F^2)$ (all data)[b]	0.1039	0.1901
Largest diff. peak/hole, $e \cdot Å^{-3}$	0.439 / -0.266	0.785 / -0.409

[a] $R_1 = \sum|F_o - |F_c||/\sum(F_o)$; [b] $wR_2 = (\sum[w(F_o^2 - F_c^2)^2]/\sum[w(F_o^2)^2]^{½}$

Table S2. Characteristics of (3,-1) bond critical points associated with the intramolecular interactions of SMe group (interatomic distance r in Å, electron denity at BCP ρ_c in a.u., energies in kcal·mol^{-1})

	8,8'-isomer			4,7'-isomer			4,4'-isomer			
	cisoid	*gauche*	*transoid*	*cisoid*	*gauche*	*transoid*	*cisoid1*	*cisoid2*	*gauche1*	*gauche2*
ΔE	10.3	3.7	0.0	12.4	8.1	8.5	13.0	10.7	4.7	10.2
ΔG^{298}	10.6	4.0	0.0	12.1	8.4	9.2	13.2	10.3	5.0	10.8
**Interactions with S atom**										
Type	**S···S**	**CH···S**	**CH···S**	**CH···S**	**CH···S**	**S···HB**	**S···HB**	**CH···S**	**CH···S**	**S···HB**
R	3.381	2.656	2.722	2.602	2.528	2.558	2.903	2.575	2.600	2.930
ρ_c	0.013	0.012	0.011	0.012	0.014	0.014	0.008	0.014	0.012	0.008
E_{BCP}	-1.7	-2.0	-1.8	-3.0	-2.6	-2.5	-1.2	-2.4	-2.2	-1.1
Type		**CH···S**	**CH···S**	**S···HB**	**CH···S**	**S···HB**	**S···HB**	**CH···S**	**CH···S**	**S···HB**
R		2.658	2.722	2.889	2.982	2.558	2.904	2.575	2.600	2.931
ρ_c		0.012	0.011	0.010	0.007	0.014	0.008	0.014	0.012	0.008
E_{BCP}		-2.0	-1.8	-1.5	-1.1	-2.5	-1.2	-2.4	-2.2	-1.1
Type			**CH···S**	**S···S**	**S···HB**		**S···HB**	**S···HB**	**CH···S**	**S···S**
R			2.740	3.526	2.820		3.044	3.022	2.902	3.416
ρ_c			0.010	0.009	0.009		0.008	0.007	0.008	0.013
E_{BCP}			-1.6	-1.2	-1.4		-1.1	-1.1	-1.3	-1.6
Type			**CH···S**				**S···HB**	**S···HB**	**CH···S**	
R			2.740				3.044	3.022	2.902	
ρ_c			0.010				0.008	0.007	0.008	
E_{BCP}			-1.6				-1.1	-1.1	-1.3	
**Interactions of CH_3 group (CH···HB)**										
R	2.318	2.472	2.490	2.417	2.517	2.828[a]		2.325	2.342	2.407
ρ_c	0.008	0.006	0.006	0.007	0.006	0.008		0.008	0.007	0.008
E_{BCP}	-1.3	-0.9	-0.8	-1.0	-0.8	-1.1		-1.2	-1.2	-1.1
R	2.318	2.438	2.473	2.294	2.335	2.828[a]		2.464	2.530	2.407
ρ_c	0.008	0.006	0.006	0.008	0.007	0.008		0.006	0.005	0.008
E_{BCP}	-1.3	-0.9	-0.8	-1.3	-1.2	-1.1		-0.9	-0.7	-1.1
R		2.473	2.473		2.863[a]			2.326	2.341	
ρ_c		0.006	0.006		0.007			0.008	0.007	
E_{BCP}		-0.9	-0.8		-1.0			-1.2	-1.2	
R		2.437	2.490					2.465	2.530	
ρ_c		0.006	0.006					0.006	0.005	
E_{BCP}		-0.9	-0.8					-0.9	-0.7	

[a] BH···C(Me) interaction
